# Comprehensive Update on Carotenoid Colorants from Plants and Microalgae: Challenges and Advances from Research Laboratories to Industry

**DOI:** 10.3390/foods12224080

**Published:** 2023-11-10

**Authors:** Delia B. Rodriguez-Amaya, Patricia Esquivel, Antonio J. Meléndez-Martínez

**Affiliations:** 1 Department of Food Science and Nutrition, Faculty of Food Engineering, University of Campinas, Campinas 13083-862, SP, Brazil; 2 Centro Nacional de Ciencia y Tecnología (CITA), Universidad de Costa Rica, San José 11501, Costa Rica; patricia.esquivel@ucr.ac.cr; 3 Escuela de Tecnología de Alimentos, Universidad de Costa Rica, San José 11501, Costa Rica; 4 Food Colour and Quality Laboratory, Facultad de Farmacia, Universidad de Sevilla, 41012 Sevilla, Spain; ajmelendez@us.es

**Keywords:** plant-derived colorants, microalgal carotenoids, microencapsulation, nanoencapsulation, green extraction, by-product valorization, regulation, safety concerns

## Abstract

The substitution of synthetic food dyes with natural colorants continues to be assiduously pursued. The current list of natural carotenoid colorants consists of plant-derived annatto (bixin and norbixin), paprika (capsanthin and capsorubin), saffron (crocin), tomato and gac fruit lycopene, marigold lutein, and red palm oil (α- and β-carotene), along with microalgal *Dunaliella* β-carotene and *Haematococcus* astaxanthin and fungal *Blakeslea trispora* β-carotene and lycopene. Potential microalgal sources are being sought, especially in relation to lutein, for which commercial plant sources are lacking. Research efforts, manifested in numerous reviews and research papers published in the last decade, have been directed to green extraction, microencapsulation/nanoencapsulation, and valorization of processing by-products. Extraction is shifting from conventional extraction with organic solvents to supercritical CO_2_ extraction and different types of assisted extraction. Initially intended for the stabilization of the highly degradable carotenoids, additional benefits of encapsulation have been demonstrated, especially the improvement of carotenoid solubility and bioavailability. Instead of searching for new higher plant sources, enormous effort has been directed to the utilization of by-products of the fruit and vegetable processing industry, with the application of biorefinery and circular economy concepts. Amidst enormous research activities, however, the gap between research and industrial implementation remains wide.

## 1. Introduction

The world’s food color market continues to be dominated by artificial color additives but driven by concerns about possible adverse health effects, their substitution with those obtained from natural sources has been widely advocated. Research and commercial interest in natural colorants have intensified with mounting scientific evidence for their health benefits, along with consumers’ demand for food to be as natural as possible [1,2]. The transition from synthetic to natural colorants, however, is not easily accomplished, considering that the latter are usually unstable, more costly, and not as easily utilized. Moreover, they have weaker tinctorial strength, a limited range of hues, and may interact with other food components [3,4,5]. Research on natural colorants should therefore focus on obtaining a wider variety of colors, using pigments with health benefits, increasing shelf life, and lowering production costs [6].

Cheaper and faster to produce, carotenoids in the market (e.g., β-carotene, astaxanthin, canthaxanthin, zeaxanthin, and β-apo-8′carotenol) are mostly products of chemical synthesis [7]. However, some natural carotenoid colorants produced from plant sources and by microbial fermentation are also available, and demand for these products is increasing.

Aside from the provitamin A activity, carotenoids have been associated with a reduced risk of developing certain types of cancer, cardiovascular diseases, cataracts, and macular degeneration [8,9,10]. More recently, other health-promoting effects have been attributed to these bioactive compounds, such as maintenance of cognitive functions [11,12,13], reduced level of depressive symptoms [14,15], and reduced risk of osteoporosis and fracture [16,17,18].

Carotenoids, however, pose daunting challenges to researchers and food processors due to their instability, lack of solubility in water, and low bioavailability [19]. Research has therefore been directed to addressing these problems, whether the carotenoids are inherent constituents of foods or are added as colorants.

The present review is intended to comprehensively update the status of natural carotenoid colorants to help researchers identify research gaps and to promote the scaling up of research findings to the industrial level. The focus is on publications in the last decade, but important research results from previous years are included.

The review initially discusses the carotenoid chromophore, then topics related to colorants derived from higher plants (commercially available colorants, encapsulation, valorization of the by-products from the fruit and vegetable processing industry), followed by microalgal carotenoid colorant, and finally regulation and safety concerns. 

## 2. The Carotenoid Chromophore

**Figure 1:**
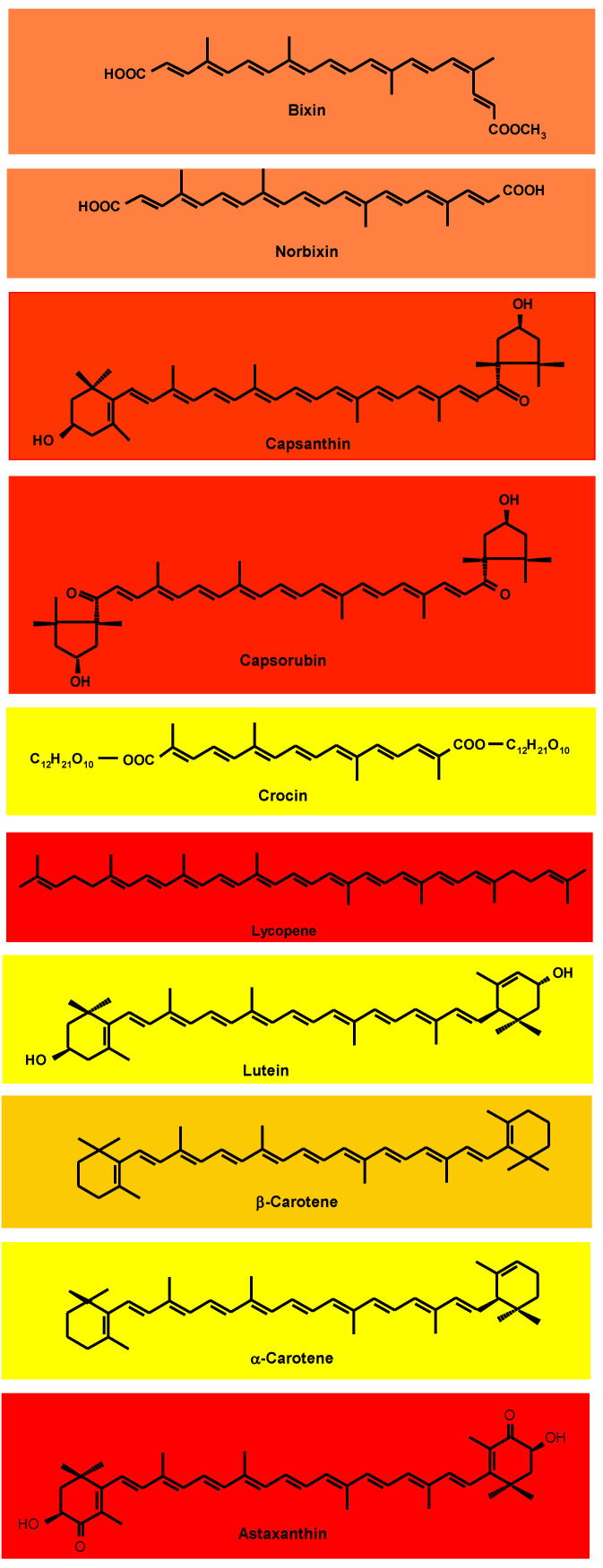
Structures of the principal carotenoids of commercial natural carotenoid colorants.

The vast majority of carotenoids absorb maximally in the wavelength range of 400–500 nm. The centrally located conjugated double-bond system constitutes the light-absorbing chromophore that confers the carotenoid’s attractive color [19]. At least seven conjugated double bonds are needed for a carotenoid to have perceptible color (faint yellow). As the number of conjugated double bonds increases, the color changes from yellow to orange, and then to red. The acyclic crocin with nine double bonds is yellow and the acyclic bixin, having a total of eleven conjugated double bonds, is orange–red (Figure 1). One of the double bonds of bixin is in the *Z*(*cis*)-configuration; the absorption maxima of *Z*-double bonds are slightly shifted to shorter wavelengths. The acyclic lycopene, with 11 conjugated *E*(*trans*)-double bonds, is red. Cyclization takes the π electrons of the ring double bond out of plane with those of the polyene chain. Thus, although also possessing 11 conjugated double bonds, β-carotene is yellow–orange because two of the double bonds are in β-rings. Lutein and α-carotene with 10 conjugated double bonds, one of which is in a ring, are yellow.

The red capsanthin has a conjugated double bond system consisting of the carbonyl group double bond, nine double bonds in the polyene chain, and one in the β-ring (totaling eleven conjugated double bonds) (Figure 1) [19]. Also red, capsorubin’s chromophore consists of nine conjugated double bonds in the polyene chain extended by the double bonds of two carbonyl groups. Red astaxanthin has nine conjugated double bonds in the polyene chain, extended by two double bonds in β-rings and 2 C=O.

On exposure to light, heat, and acids, carotenoids undergo *E-Z* isomerization, which results in a small spectral shift to shorter wavelengths, thus a slight lightening of the color [19].

Apart from the chemical structure, the color of carotenoids is also dependent on other factors such as concentration and aggregation or interaction with proteins. Indeed, carotenoproteins extend the colors of carotenoids to blue, purple, green, and brown [20].

The conjugated double-bond system is also responsible for the susceptibility of carotenoids to oxidation [19]. Color fading is a well-known problem during processing and storage of carotenogenic foods. As oxidative degradation advances, the conjugated double bond system is progressively shortened until the number of conjugated double bonds falls below seven and the carotenoid molecule becomes colorless [19,21,22].

## 3. Carotenoid Colorants Derived from Higher Plants

**Figure 2:**
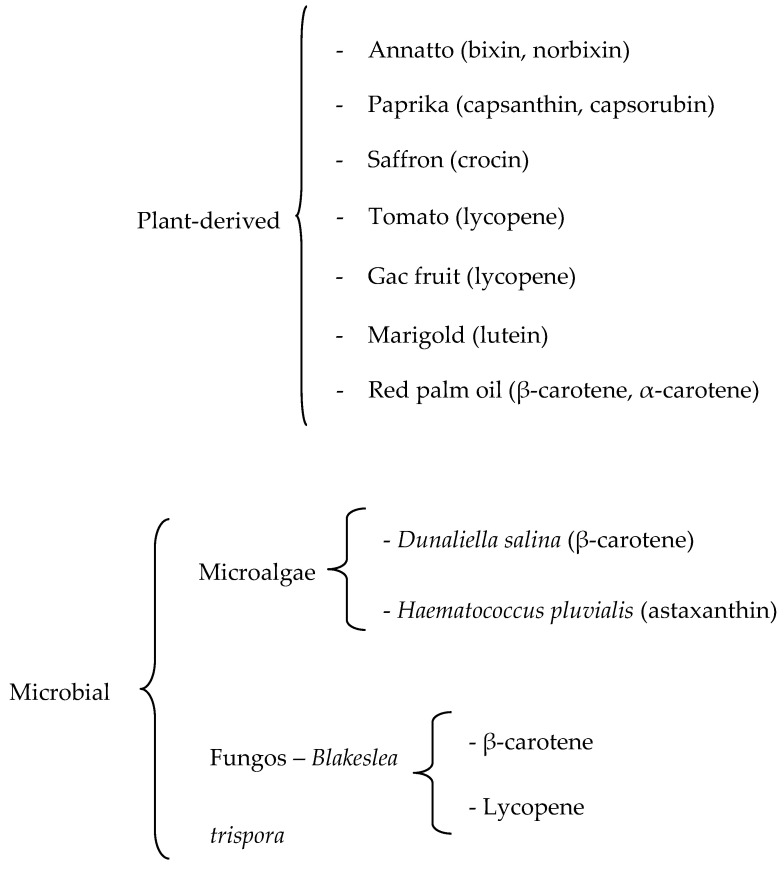
Summary of commercially available natural carotenoid colorants for food use.

Recent investigations on plant-derived colorants focus on: (a) optimizing extraction to obtain the maximum pigment yield and to turn to green extraction, (b) stabilizing the colorant by microencapsulation or nanoencapsulation, and (c) additional benefits (aside from color) of the main coloring carotenoids, especially health promoting effects. A summary of commercially available natural colorants is presented in Figure 2.

A major problem with plant-derived carotenoid colorants is batch-to-batch variation in the carotenoid concentrations of the raw materials due to cultivar/varietal differences, seasonal and geographic variability, maturity at harvest, climatic conditions, etc. [19]. According to Bogacz-Radomska and Harasym [23], the main disadvantages of the production of carotenoid colorants from plant materials are the high cost, geographic determinants, and seasonality of the raw material. 

It is now widely accepted that the reduction of the risk of chronic diseases by plant foods is not due to a single food component or a single class of compounds but to the coexistence of different bioactive compounds [19]. They have different modes of action, act at different stages of disease development, and demonstrate additive or even synergistic effects. Thus, plant extracts used as colorants, which usually contain bioactive compounds other than the carotenoids, can be more beneficial for preventing the risk of some chronic diseases than individual carotenoids.

### 3.1. Annatto Bixin and Norbixin

The orange-to-red annatto is derived from the resinous, thin seed coats of the capsular fruits of *Bixa orellana*, a tropical tree believed to be native to Central and South America [19]. Annual world production of the seeds is approximately 14,500 tons dry weight (DW) [24]. Two-thirds of the production is commercialized as dried seeds and the rest as colorants. Latin America produces 60% of the total world production, followed by Africa (27%) and Asia (12%). The main producers in Latin America are Peru, Brazil, and Mexico.

Annatto is available as an oil-soluble extract, water-soluble extract, suspension, emulsion, encapsulated product, and dried powder. It is used worldwide to color a wide range of products, such as butter, cheese, margarine, mayonnaise, sauces, salad dressings, mustard, soups, juices, ice cream, bakery products, snacks, soft drinks, desserts, meat products, and macaroni [19].

The coloring agent in oil-soluble annatto preparations is bixin, a monomethyl ester of a dicarboxylic apocarotenoid (carotenoids in which the carbon skeleton has been shortened by removal of fragments from one or both ends of the usual C-40 structure) (Figure 1) [19]. Hydrolysis (saponification) liberates the dicarboxylic, water-soluble norbixin. 

Unlike most carotenoids, which occur in nature in the all-*E*-configuration, bixin is normally in the *9Z*-form (Figure 1). In general, carotenoids undergo *E-Z* isomerization and oxidative degradation during processing and storage of foods [19,21,22]. The latter consists of epoxidation, cleavage to apocarotenoids, and finally, cleavage to low-mass (volatile) compounds. On exposure of bixin to light, heat, or acids during extraction, processing, and storage, *Z-E* isomerization occurs, slightly accentuating the red color. Bixin’s conjugated double bond system is also prone to oxidative degradation, eventually leading to its cleavage to low-mass (volatile) compounds [25], manifested by the loss of color and sometimes by off-flavor. 

It is well known that bixin is unstable in the presence of oxygen, light, high pH (alkali), and heat. Light, reduced pH, and metal ions, with and without H_2_O_2_, increase the bleaching of norbixin, whereas chelators and the natural antioxidants, ascorbic acid and tocopherol, reduce the bleaching [26]. 

Norbixin in buffered aqueous solutions was stored under light and in the dark and analyzed by mass spectrometry [27]. Compounds with both higher and lower masses than norbixin were detected, suggesting that oxidation products and oxidative cleavage products of norbixin were produced. The compounds formed were not identified, however. The norbixin concentration decreased during storage, the loss occurring faster under light. 

The effects of different extraction methods on the degradation of bixin and the formation of undesirable volatile compounds were investigated by Chuyen and Eun [28]. Extraction with sodium hydroxide solution at 50 °C was recommended for extracting sufficient pigment from annatto seeds with minimum risk of forming harmful volatile compounds. Extraction with acetone in a Soxhlet extractor resulted in the highest bixin yield but produced the highest amount of xylene. Extraction with soybean oil at 120 °C gave the lowest bixin yield and caused significant bixin degradation. All three methods produced negligible amounts of toluene. 

The impact of solvents (ethyl acetate, methanol, and ethanol) and varying process parameters such as time (10, 20, and 30 min), temperature (50, 60, and 70 °C), and seed-to-solvent ratio (1:5, 1:10, and 1:15) on bixin yield was evaluated by Jayakumar et al. [29]. Methanol was observed to be the optimal solvent; the optimum conditions were 70 °C, a seed-to-solvent ratio of 1:15, and a treatment time of 30 min.

Microwave dielectric heating and ultrasonic cavitation bubbles demonstrated substantial improvement in the extraction of carotenoids over conventional heating methods, with microwave heating being the optimal technique [30]. Using the optimum extraction parameters for annatto seeds (at least 30 min extraction with ethyl acetate, solvent/material ratio of 0.05 L/g), the yield of bixin reached about 85% with sonication and 95% with microwave heating. 

Extraction obtained by using ultrasound (UAE) is mainly attributed to the effect of acoustic cavitations produced in the solvent as a result of ultrasound wave passage [31]. Compared to other extraction techniques such as microwave-assisted extraction (MAE) and supercritical fluid extraction (SFE), the ultrasonic device is less expensive and much easier in practice. UAE was found to be a more efficient process compared to conventional extraction. 

Physical separation methods were used to obtain the pigment of semi-defatted annatto seeds, the residue produced after the extraction of the tocotrienol-rich oil using SFE [32]. The physical methods included mechanical fractionation and an integrated process of mechanical fractionation and low-pressure solvent extraction. The latter method required a significantly higher cost. The mechanical fractionation method was considered an adequate and low-cost process to obtain a pigment-rich product from semi-defatted annatto seeds.

Norbixin is water-soluble at neutral and alkaline pH but starts to precipitate below neutral pH [33]. The addition of whey protein isolate prevented norbixin precipitation between pH 2 and pH 7, except at pH 5. At the latter pH, isoelectric precipitation of whey protein isolate was prevented by the inclusion of alginate. Encapsulation of norbixin within liposomes was also shown to increase its water dispersibility and chemical stability under acidic pH conditions [34].

Annato extract, apart from being a colorant, has other desirable properties such as antimicrobial and antioxidant activities. The survival curve of the food-born pathogen *Escherichia coli* in mayonnaise with annatto reached zero during 15 and 12 days after inoculation at 4 and 25 °C, respectively [35]. All annatto extracts obtained by maceration with distilled water at various pH and temperature had the potential to inhibit *E. coli* and *Staphylococcus aureus* [36]. Paprika, lutein, and especially annatto, investigated against ten microorganisms, had antimicrobial effects, particularly against gram-positive bacteria, including *Staphyloccus aureus*, *Staphylococcus epidermidis*, *Bacillus cereus*, *Bacillus subtilis*, *Listeria monocytogenes*, and *Streptococcus pyogenes* [37]. The addition of 1 to 10% annatto to bread formulation adequately inhibited the growth of *Aspergillus niger*, *Neurospora sitophila*, *Rhizopus stolonifer*, major pathogens and spoilage microorganisms of bread and many other food products [38]. Antioxidant activity was also observed. The bread with annatto had a longer shelf life and acceptable sensory qualities.

β-carotene, α-carotene, lycopene, β-cryptoxanthin, lutein, and zeaxanthin have been the most studied carotenoids in terms of human health. Epidemiological and supplementation studies have been carried out and submitted to meta-analyses [10]. The health benefits of bixin and norbixin have not been the focus of research. 

In several pathologies, erythrocytes exhibit high susceptibility to hemolysis because of the oxidation of cellular components. Beni et al. [39] investigated whether food-grade annatto carotenoids could increase human erythrocyte resistance to hemolysis in vitro and ex vivo. For the in vitro experiment, erythrocytes from healthy volunteers were isolated and co-incubated with bixin or norbixin and 2,2′-azobis(2-amidinopropane) dihydrochloride, glucose, or sodium nitrite as hemolysis inducers. In the ex vivo study, healthy volunteers consumed a capsule containing bixin or norbixin or placebo for 7 days before blood sample collection. The results supported the hypothesis that supplementation with annatto carotenoids exerted antihemolytic properties by preventing the oxidative damage of human erythrocytes.

Bixin and crude extract were examined in vitro in human lung cancer, cervical cancer, and breast cancer cells [40] Anti-proliferative activity appeared promising on both the isolated pigment and the crude extract. 

In a randomized, controlled crossover study involving 12 healthy subjects, the effect of annatto intake associated with a single high-caloric meal (high fat and high carbohydrate) was evaluated [41]. Norbixin intake did not affect biochemical blood markers but reduced the postprandial levels of inflammatory cytokines and lipid oxidation 60–120 min after the meal. Bixin only partially prevented postprandial-induced lipid oxidation. The results indicated that the intake of norbixin might be an alternative to reduce the postprandial inflammatory and oxidative stress responses to high-caloric meals.

### 3.2. Paprika Capsanthin and Capsorubin 

A possible problem with plant-derived colorants is that the flavor of the plant source may be carried over to the final product and may not be compatible with it [42]. This is not the case with paprika and saffron, however, both of which serve as spice and colorant. 

Paprika is a deep red, pungent powder obtained from red pepper (*Capsicum annuum*) pods. It has a complex mixture of carotenoids, the most prominent of which are capsanthin and capsorubin (Figure 1) [19].

Paprika oleoresin is produced by solvent extraction of the ground powder [19]. Solutions in edible vegetable oil and water-miscible forms of oleoresin are also available in the market. Paprika is limited to products compatible with its flavors, such as meat products, sausages, smoked pork, sandwich spreads, soups, sauces, salad dressings, spice mixtures, cheeses, orange juice, snacks, confectionery, and baked products. 

Paprika powders from Bulgaria, China, Hungary, Peru, Serbia, and Spain were examined to identify the most important differences in their major characteristics and to try to find chemical components that reveal their origin [43]. Carotenoids were found at the highest concentrations in samples from Peru and Spain and at the lowest in Serbian samples.

Compared with the traditional extraction methods, UAE and MAE can improve the color value of paprika pigment and shorten the extraction time [44]. More importantly, it can avoid the decomposition of active substances caused by long-time extraction under high temperatures and pressure. 

Paprika oleoresin was obtained by accelerated solvent extraction (ASE), maceration extraction, and UAE [45]. ASE, also known as pressurized liquid extraction (PLE) or pressurized fluid extraction, is an automatic extraction technology performed at elevated temperature and pressure to achieve efficient extraction of compounds from solid or semisolid samples in a very short time. The color values, total carotenoid, and capsaicinoid content were significantly higher for ASE than the other two extraction methods. 

Cinnamaldehyde and carotenoids in cinnamon and paprika oleoresins, respectively, exhibited pronounced antimicrobial and antioxidant potential [46]. The coencapsulation of the two oleoresins by spray chilling promoted greater stability and synergism between them. Cinnamon:paprika (1:1 and 2:1) mixtures showed a synergistic effect against *Penicillium paneum* and *Aspergillus niger*. The extracts also prevented the growth of microorganisms without direct contact with the agar. The concentration of carotenoids in the particles remained constant throughout the 49 days of storage at 5 and 25 °C.

De Aguiar et al. [47] reviewed the application of supercritical fluid technologies to *Capsicum* peppers and derived products, including oleoresin. Trends in encapsulation technologies applied to *Capsicum* and derivatives were also discussed. However, the compounds of interest were capsaicin and phenolic compounds, not carotenoids. 

To offer consumers healthier meat products, paprika oleoresin was used to replace or reduce the nitrite level [48]. Approximately 3/4 of the initial nitrite level could be replaced with 0.1% paprika oleoresin solution.

### 3.3. Saffron (Crocin)

Saffron, the dried stigmas of *Crocus sativus* flowers, is considered the world’s most expensive spice. *C. sativus* has been in cultivation for many centuries. Presently, the major growing areas are Iran, India, Morocco, Spain, Turkey, Italy, Afghanistan, and New Zealand [49]. Iran produces almost 90% of the total world production. Saffron is used in the food industry for the manufacture of a wide range of products, including dairy, bakery, sauces, soups, chicken, rice, and beverages [19,42]. 

Kotheri et al. [49] called attention to the fact that only the stigma of the flower is used, the remaining floral parts going to waste. Utilization of this discarded material certainly merits research.

Saffron’s prominent constituents are crocin, picrocrocin, and safranal, responsible for its color, bitter taste, and aroma, respectively [50]. These compounds are the bio-oxidative cleavage products of zeaxanthin. The yellow crocin is a crocetin digentiobiose ester. It is a symmetrical apocarotenoid in which the two carboxylic groups of the C-20 carotenoid crocetin are esterified with the disaccharide gentiobiose (Figure 1), making the pigment water-soluble.

Crocin is a highly bioactive compound, but its use is limited by its instability to pH variations, light, heat, and oxidative stress, along with rapid absorption and low bioavailability [51]. Encapsulation of saffron extracts within polymeric matrices has been shown to improve their stability during storage [52] and in simulated gastric conditions [53]. 

Numerous health-promoting activities have been attributed to saffron/crocin, such as antioxidant, antitumor, anti-inflammatory, anticancer, antidiabetic, anxiolytic, neuroprotective, learning and memory-enhancing, antidepressant, antihyperlipidemic, antiatherosclerotic, anti-ischemia, antigenotoxic, hypoglycemic, hypotensive, antidegenerative [50,51,54,55,56,57,58,59,60,61,62]. Notably, intense research on the health effects of crocin has been undertaken, not in food research, but in pharmacological studies. The demand for saffron has been rising in the pharmaceutical industry [56] 

Crocin and other crocetin glycosides are also found in the fruit of *Gardenia jasminoides* and *Gardenia augusta* [63]. Gardenia yellow is a colorant produced by water or ethanol extraction of these fruits and is approved for food use in Japan and China, but not in the USA or the EU.

### 3.4. Tomato and Gac Fruit Lycopene 

The widely reported health benefits of lycopene and lycopene-rich tomatoes, especially in relation to prostate cancer, had drawn worldwide attention. Hence, tomato lycopene extract and tomato lycopene concentrate as oleoresin, powder, and water-dispersible preparations became commercially available. 

Interest in lycopene heightened with the comprehensive studies on inverse associations between tomato or lycopene intake or serum lycopene level and the risk of prostate cancer [64,65,66,67,68]. Lycopene was also linked with a reduction in the risk of developing other types of cancer. In a large prospective analysis with 20 y of follow-up, women with high plasma carotenoids were found to be at reduced breast cancer risk, particularly for more aggressive and ultimately fatal disease [69]. Meta-analyses also associated lycopene with a lower risk of esophageal cancer [70] and oral and pharyngeal cancer [71].

There is also good evidence from meta-analyses for an association between intake and/or blood concentration of lycopene with a reduced risk for stroke and cardiovascular diseases [72,73,74], A comprehensive meta-analysis suggested that high-intake or high-serum concentration of lycopene was linked with significant reductions in the risk of stroke (26%), mortality (37%) and cardiovascular diseases (14%) [75]. Moreover, supplementation with tomato products and lycopene had positive effects on blood lipids, blood pressure, and endothelial function [76].

The Asian gac fruit (*Momordica cochinchinensis*), has been shown to have an impressively high content of lycopene, 408 μg/g fresh weight (FW) according to Vuong et al. [77]. Traditionally used in Asia to provide red color for cuisines and enhance vision health, it is now commercially available as gac powder and gac oil, manufactured as natural colorants and medicinal supplements [78].

### 3.5. Marigold Lutein

Marigold (*Tagetes erecta*) flower is the commercial source of lutein. Originally cultivated in Mexico and other warmer areas of America, marigold is now naturalized in other tropical and subtropical regions [79].

Marigold lutein has been used as an additive in poultry feed to improve the pigmentation of the bird’s fat, skin, and egg yolk [16]. The main coloring component is all-*E*-lutein esterified with fatty acids. 

Lutein is highly beneficial in terms of human health. According to most epidemiological and clinical trials, lutein and zeaxanthin have a role in the prevention of certain eye diseases such as age-related macular degeneration, cataracts, and retinitis pigmentosa [80]. 

A meta-analysis of six longitudinal cohort studies showed that dietary intake of lutein and zeaxanthin was significantly related to reduced risk of late age-related macular degeneration, but not to reduced risk of early age-related macular degeneration [81]. Statistically inverse association was observed between intake of these carotenoids and neovascular age-related macular degeneration. Another meta-analysis showed significant benefits of lutein and zeaxanthin supplementation to visual acuity and contrast sensitivity for macular degeneration patients, positively associated with elevation of macular pigment optical density [82].

Blood levels of lutein and zeaxanthin were inversely associated with age-related cataracts [83] and nuclear cataracts [84]. Likewise, dietary intakes of these carotenoids had a significant inverse association with nuclear cataracts and posterior subcapsular cataracts, but not with cortical cataracts [85].

Meta-analyses have also shown that higher intake and/or blood concentration of lutein/zeaxanthin were associated with lower risk of some types of cancer, such as esophageal cancer [70] and non-Hodgkin lymphoma [86]. Moreover, these carotenoids have also been linked with cardiometabolic health [87] and better cognitive function [10,11,88].

### 3.6. Red Palm Oil α- and β-Carotene

For a long time, orange carrot was the popular source of the provitamin A carotenoids β-carotene and α-carotene. A more concentrated source of these two carotenoids is red palm oil, which has been investigated and proposed for food-based intervention for vitamin A deficiency amelioration [89].

Oil palm (*Elaeis guineenses*) has been cultivated in Asia and Central America. It is the most important economic crop in Malaysia; other leading palm oil-producing countries are Indonesia and Thailand [90].

Red palm oil can be obtained from the mild processing of crude palm oil while refined, bleached, and deodorized palm oil is obtained by physical refining or chemical refining of the crude palm oil [90]. The bleaching process that uses bleaching clay removes the color pigments and residual soaps from the oil. During the deodorization step of physical refining, edible oils are subjected to high temperatures (250–270 °C) and low pressures (3–5 torr) to remove free fatty acids and volatile compounds that affect the oil’s odor and flavor. Palm carotenoids are removed during the refining process to obtain clear oil which is better for consumer acceptance and industry purposes.

Refined, bleached, and deodorized palm oil is the major processed product, used in more than 150 countries around the world [91]. Red palm oil has become well accepted in Africa, Indonesia, India, China, and Malaysia. It is not widely available in the international markets. The minimally processed palm oil has been introduced to Western consumers only recently, receiving mixed reactions. Some people find the red–orange hue unappetizing, while others view the color as a welcome reminder of the oil’s high carotene content [92]. 

Red palm oil can be added to food products such as cooking oil, shortening, spreads, salad dressings, and margarine. [93]. One product that has the potential to be developed based on red palm oil in Indonesia is margarine because it can act as a source of fat and also provides natural coloring and high nutrition from phytonutrient components, especially its carotene content. In Brazil and African countries, red palm oil has been used for years for culinary purposes.

Red palm oil is rich in phytonutrients such as tocotrienols, tocopherols, carotenoids, and phytosterols. Aside from the provitamin A activity, other health benefits attributed to red palm oil include improvement of ocular complications; cardioprotective effects in ischemic heart disease; antiatherogenic, antihemorrhagic, antihypertensive, and anticancer properties; support of normal reproduction for both males and females; improved management of diabetes and chemotherapy; improved management of hypobaric conditions; and protection against infection [91]. However, human studies to support these claims are lacking.

Considering the large-scale production of refined, bleached, deodorized palm oil, during which enormous amounts of β-carotene and α-carotene are wasted, sporadically through the years, proposals to separate these valuable carotenoids from the oil before processing have surfaced. An example is the recent work of Hoe et al. [94]. Liquid-liquid extraction was used to directly extract palm carotenes from crude palm oil without disrupting the subsequent production of refined palm oil. Dimethyl sulfoxide and dichloromethane were adopted as solvent systems because of their excellent performance in palm carotene extraction. After optimization, the optimal percentage recovery of palm carotene was 41%. The total recovery of palm carotene increased up to 68% by using a multistage extraction approach. 

## 4. Encapsulation of Carotenoids 

Introduced primarily as a stabilizing technique for bioactive compounds, encapsulation has now been shown to provide many important benefits. These compounds are encapsulated in a polymeric wall material by means of physical or chemical processing, which can be performed at a macro- (>1000 μm), micro- (1–1000 μm) or nano- (1–100 nm) level, depending on the size of the encapsulated particles. For carotenoids, the following advantages of encapsulation have been summarized [95,96,97,98,99,100,101,102]:

- Protection of carotenoids from undesirable environmental conditions and matrix interactions, thus avoiding degradation;
- Ease and flexibility of handling;
- Better solubility, facilitating incorporation in food products;
- Controlled release and improved bioavailability;
- Suppressing undesired aroma/flavor.

Encapsulation can increase the bioavailability or bioactivity of the bioactive compound, permitting a controlled release rate at the site of action [95,100]. Formulations with microencapsulated lycopene to be used as dietary supplements are already produced commercially by LycoRed Ltd., BASF, and Roche Vitamins [103].

### 4.1. Microencapsulation 

Microencapsulation has been utilized as a strategy to reduce the degradation of carotenoids and to facilitate their incorporation into foods in aqueous matrices. Research approaches in this field are focused on studying physical and chemical processes for particle formation and stability and metabolism of encapsulated carotenoids [104,105,106]. Some techniques used are spray-drying of emulsions; spray chilling or spray cooling using fat or wax membranes; emulsification by dispersing the compound of interest in an immiscible liquid phase together with the wall material; fluidized bed coating by spraying with air solid particles through particles of the coating wall material; liposome entrapment of an aqueous phase in a single or multilayered phospholipid membrane forming vesicles; and coacervation by emulsification of the compound into a dissolved gelling protein [101,107,108,109]. 

Spray drying is the most extensively applied encapsulation technique in foods. The most widely used materials for encapsulation are polysaccharides and maltodextrins; proteins and lipids are also employed [97,101,110]. 

#### 4.1.1. Recent Studies on the Microencapsulation of Carotenoids

Various encapsulating agents such as modified starch, chitosan, inulin, Arabic gum, soy protein, and maltodextrin, have been studied [97,111,112,113]. Encapsulation methodologies are classified as physical (spray, freeze, and drum drying, processes using supercritical fluids, centrifugal extrusion, fluid bed, and pan coating), physicochemical (spray cooling, solvent evaporation extraction, hot melt coating, simple or complex coacervation, ionic gelation), and chemical (interfacial polycondensation, polymerization, and cross-linking and in situ polymerization) [97]. 

The potential of different polysaccharide mixtures as wall material for encapsulation was appraised by several authors. A marigold flower extract (containing approximately 200 mg/100 DW lutein) was obtained using SC-CO_2_ and encapsulated with a mixture of maltodextrin and Arabic gum (60:40) [114]. After spray drying, a 78% efficiency was achieved (128 mg/100 g powder). Lutein half-life was increased up to 6.9-fold compared to the native extract after 60-day storage (22 °C, in the dark, 80% relative humidity). The encapsulation of astaxanthin extracts from *Haematococcus pluvialis* was optimized using a mixture of maltodextrin (5–40 dextrose equivalent) and gelatin (ratio of 2.1:1) by spray drying [115]. The encapsulation efficiency amounted to 72%.

Microencapsulation of a mixture of acerola (*Malpighia emarginata*) and ciriguela (*Spondias purpurea*) juices (ratio 60:40) was achieved after spray-drying, using wall material mixtures of Arabic gum and maltodextrin with different levels of xanthan gum [116]. A better carotenoid encapsulation was observed when adding 0.3% xanthan gum. In another study, Arabic gum was used as wall material for microencapsulation of pressed palm oil extracts [117]. After SC-CO_2_ extraction, carotenoids were spray-dried. Microencapsulation efficiency of β-carotene reached values between 61 and 86%.

Electro-spraying was employed to encapsulate β-carotene emulsions, using two different protein solutions (whey protein concentrate and zein) and soybean oil [118]. Ultrasonication affected carotenoid contents negatively in zein protein emulsions. On the other hand, in most cases, except for unstable whey protein concentrate emulsions, the carotenoid bioaccessibility increased, making up 0.03–0.05% of total carotenoids. The microencapsulation efficacy amounted to 34% for zein emulsions and 22% for whey protein after high-speed homogenization and ultrasonication. 

Microencapsulation strategies of extracts obtained from food processing by-products have also been evaluated. Carotenoid-rich oil extracted from gac peel was encapsulated using whey protein concentrate and Arabic gum (7:3 ratio) by spray drying [119]. After 6 months of storage, 80% of carotenoids were retained. Microcapsules of carotenoid-rich extracts obtained from mango and banana peel powders and tamarillo freeze-dried pulp were prepared with maltodextrin as an encapsulating agent, obtained by spray-drying [120].

SC-CO_2_ was used for the encapsulation of carrot pomace extract with linseed oil. Sunflower wax was utilized as wall material [121]. With this technology, microparticles are obtained after the rapid expansion at atmospheric pressure of a saturated SC-CO_2_ fluid with a mixture of bioactive compounds and cell wall material. The rapid release of CO_2_ produces solid or liquid particles. The total carotenoids of the extracts came up to 200 mg/100 g DW. Encapsulation efficiency reached 92, 87, and 86%, with 10, 20, and 30 MPa, respectively. 

Annatto seed extracts were encapsulated as a source of norbixin by spray drying using Arabic gum as an emulsifying agent. The norbixin microcapsules were added to tangerine juices, thereby increasing the half-life of this compound from 393 min to 29.7 days [122]. 

Maas (*Renealmia alpinia*) pulp was encapsulated by spray drying using maltodextrin (4–7 °Brix), Arabic gum (15 °Brix), and mixtures thereof (7.5 °Brix) as wall materials [123]. The microcapsules were added to yogurt and the pigment stability was assessed during 28 days of storage. Initial total carotenoid concentration in the capsules amounted to 17, 19, and 34 mg β-carotene per 100 g powder, for maltodextrin, maltodextrin and Arabic gum mixture, and Arabic gum, respectively. A reduction in carotenoid contents was observed in all samples during storage, both at 4 and 25 °C. The maltodextrin formulation showed the highest retention (47% of the initial concentration). 

Microencapsulation of palm oil was achieved using the complex coacervation method [124]. Samples were dried both by atomization and lyophilization using chitosan:xanthan and chitosan:pectin as cell wall materials. The encapsulation efficiency was higher for lyophilized microparticles (52 and 62%, for chitosan:pectin and chitosan:xanthan, respectively), compared to spray-dried samples (22 and 33%). 

Three wall materials (starch, gelatin, and sodium lignosulfate) were compared for the encapsulation of canthaxanthin to be used as feed for laying hens [125]. The microcapsules contained 10% canthaxanthin and the microencapsulation efficiency reached 95% after spray-drying of the emulsion subjected to high-pressure homogenization. Daily, 5 mg/kg of canthaxanthin was fed to the animals. Color was improved in fresh yolks and after frying, boiling, and storage of the eggs (40-day at 4 and 25 °C). 

Microparticles loaded with norbixin were formed using spray drying and different ratios of Arabic gum and maltodextrin as carriers [126]. The highest encapsulation efficiency was obtained using 100% Arabic gum as a carrier. Encapsulation improved the stability of norbixin when subjected to thermal treatments (60, 90, 98 °C during 300 min).

Microparticles loaded with sea buckhorn extracts, rich in carotenoids, were formed using whey protein isolates as carriers and acacia gum [127]. Both coacervation and freeze drying were employed. Powders with 3 mg total carotenoids per g were obtained and used for muffin preparations (2 mg/100 g total carotenoids). After 21-day storage (25 °C), a 55% decrease in carotenoid contents was observed in the muffins.

Stability towards UVA–visible light of spray-dried microencapsulated bixin powders was evaluated for 30 days [128]. The encapsulating materials were sucrose, maltodextrin, and mixtures including gum Arabic and pectin as encapsulation enhancers. A correlation was observed between encapsulation efficiency and improved photostability of the colorant. The observed degradation was 30–70 times slower compared to that of unprotected colorants. The encapsulation of annatto extract was assessed by ionic gelation using quinoa, lentil, and soy proteins, and sodium caseinate as carrier materials [129]. The encapsulation efficiencies ranged from 58% to 80%; thus, more studies were deemed necessary to improve the encapsulation efficiencies. Commercial and solvent-extracted annatto were microencapsulated using modified starch and gelatin blend as wall material by spray drying [130]. The highest encapsulation efficiency (86%) was recorded for microcapsules prepared with modified starch:gelatin ratio of 60:40. 

A hydrocolloid extracted from psyllium husk was used as an encapsulating agent of an annatto extract [131]. The microencapsulated extract added to the ice cream formulation improved color homogeneity and provided superior emulsion stability over the product’s shelf life.

Using gum Arabic and maltodextrin in different proportions as wall materials, the microencapsulation by spray-drying improved the thermal stability of norbixin [126]. The activation energy required for the degradation of norbixin microcapsules (Ea = 15.08 kcal/mol) was twice as high as that required for unencapsulated norbixin. 

Norbixin microcapsules were added to isotonic tangerine soft drinks in a quantity not exceeding food additive regulations, the addition favorably affecting beverage stability during storage under accelerated conditions (heat and light) [122].

The stability of bixin in lipid-core nanocapsules during photosensitization (5–25 °C) and heating (65–95 °C) in model systems of ethanol:water was evaluated [132]. The activation energies for the nanocapsules were superior to those of free bixin in both the photosensitization and heating experiments, suggesting that encapsulation increased the stability of bixin.

Addressing low water solubility, aqueous nanodispersions of annatto extracts were prepared using a simple organic solvent-free and low-energy method [133]. The nanodispersions were prepared by the addition of water to a mixture of extract and surfactant(s). The best results were achieved using polysorbate 80/sorbitan monooleate (diluted in water, 1:10).

Gum Arabic, modified starches, and their combination (1:1) were used as wall materials in the microencapsulation of paprika oleoresin by emulsification followed by spray drying [134]. Powders had low values of aw (water activity) and moisture content, high solubility, and good encapsulation efficiency (>90%). Octenyl succinic anhydride modified-waxy rice starch was found to be a good wall material to protect lipophilic carotenoids [135]. The pigment powder encapsulated by freeze-drying had higher color stability and better emulsion and heating stabilities than that encapsulated with spray drying and the native pigment.

It was observed that water/oil/water (W/O/W) multiple emulsion stabilized by sequential adsorption of whey protein/and pectin was the most efficient technique. resulting in better encapsulation efficiency for crocin, picrocrocin, and safranal [136]. 

Encapsulating in chitosan–sodium alginate nanoparticles prepared by a modified ionic gelation method enhanced the stability of crocin [137]. Nanoencapsulation of crocin with casein effectively improved the storage, thermal, and lighting stability of crocin [138]. For the pharmacokinetic study, animals treated with crocin/casein nanocomplexes showed significantly higher serum crocin levels than those treated with crocin solution. 

Spray drying conditions were optimized for the encapsulation of gac oil using a blend of whey protein and gum Arabic as the wall material [139]. The powder obtained, containing a high content of unsaturated fatty acids, β-carotene, and lycopene, had an attractive red–yellow color and could be used as a nutrient supplement and natural food colorant.

Progressive degradation of color, β-carotene, and lycopene was observed in encapsulated gac oil powders with increasing storage temperatures and storage times [140]. However, the degradation was much less when the encapsulated powder was stored at low temperatures in the absence of air and light. The results also showed that the encapsulated gac oil powder could be successfully incorporated into food products (yogurt, pasteurized milk, cake mix).

#### 4.1.2. Effects of Microencapsulation on Carotenoid Absorption

The effect of microencapsulation on the bioavailability and bioaccessibility of carotenoids has also been studied considering their application in food [141]. Bioaccessibility of a commercial β-carotene water-dispersible powder was compared to that of two forms of encapsulated β-carotene, one spray-dried with maltodextrin and the other was a chitosan-coated β-carotene alginate [142]. Encapsulation efficiencies reached 38 and 55% for maltodextrin powder and chitosan-alginate beads, respectively. The water-dispersible powders showed the highest release of β-carotene (93%) and the highest incorporation in micelles (36%). When added to a food matrix, the release and micelle incorporations were 35 and 17% with pudding and 27 and 5.5% in yogurt, respectively. 

The bioaccessibility of astaxanthin microcapsules was studied in pickering emulsions, stabilized after spray drying with lupin protein-based aggregates [143]. A microencapsulation efficiency of 90% was observed when adding 6% of protein aggregates for emulsification. A bioaccessibility of 80% was reached after encapsulation, 5-fold higher than that of free astaxanthin with 16%. After 4 weeks of storage at 25 and 45 °C, better retention of astaxanthin was observed in samples with higher lupin protein aggregate contents.

The improvement of the bioavailability of esterified astaxanthins was observed after microencapsulation using electrostatic complexation of whey protein and Arabic gum [144]. It was compared to the non-encapsulated mixture avoiding the complex coacervation by not adjusting the pH to 4.0. The in vitro release was 26%, compared to the control samples with 15%. The astaxanthin level in the plasma of BALC/c mice after a single-dose oral gavage (100 mg astaxanthin esters per kg) was 2-fold higher than that obtained with encapsulated samples.

The stability and in vitro release of encapsulated fucoxanthin were studied [145]. Fucoxanthin from *Undaria pinnatifida* was encapsulated by spray drying, using maltodextrin, Arabic gum, pea protein isolate, whey isolate, and gelatin as wall materials. Maltodextrin, Arabic gum, and whey were found to increase the stability of this carotenoid. 

The effect of freeze-drying and spray-drying on carotenoid in vitro release at the intestinal level was compared. Soy protein isolate was used as wall material for encapsulation of red pepper waste extracts. A release of 81% after 4 h was observed after freeze-drying, while 68% was observed after 6 h after spray-drying [146]. 

### 4.2. Nanoencapsulation

Nanoparticles have also been developed to protect carotenoids against degradation and improve bioavailability. There are different types of nanostructures, such as nanoemulsions (W/O, O/W, O/W/O, W/O/W), nanoprecipitates (where the organic carrier of the compound of interest is emulsified and the polymer is precipitated, diffusion of the organic solvent occurring in the aqueous medium), hydrogel particles and filled hydrogel particles, cyclodextrin, nanofibers, nanoliposomes, casein micelles, solid-lipid nanoparticles (where bioactive compounds are encapsulated in a solid lipid matrix by congealing), lipid carriers, and polymeric nanoparticles [104,109,110,147,148,149]. Techniques such as nanoprecipitation, coacervation, inclusion complexation, and supercritical fluid encapsulation correspond to self-organization and self-assembly of molecules. They are affected by ionic strength, concentration, and pH. Extrusion, homogenization, electrospinning/spraying, nano spray drying, and emulsification-solvent evaporation may also be used [150].

#### 4.2.1. Recent Studies on the Nanoencapsulation of Carotenoids

Applications of biopolymeric nanocarriers have been reviewed. Some of the studied nanocarriers are zein, alginate, whey protein and whey protein isolate and hydrolysate, chitosan, pectin, gelatin, sodium, and calcium caseinate, soybean protein isolate, modified starch, and modified short glucan chains [7,150]. 

Different strategies have been used to encapsulate β-carotene in nanostructures. Ultrasonic homogenization was studied to produce nanoemulsions as carriers of β-carotene. Different surfactant levels (Tween 80 and soya lecithin) were evaluated to optimize emulsifying conditions that affected droplet size and β-carotene stability. An 86% β-carotene retention was achieved after one week of storage. The best results were observed using 5.8% surfactant and 6.5% olive oil [151]. β-carotene was also encapsulated in an O/W nanoemulsion using high-pressure dual channel microfluidization, with quillaja saponins and whey protein isolate as emulsifiers [152]. Although the emulsions remained stable after 14 days of storage at 4 and 25 °C, carotenoid degradation increased with higher storage temperatures. Pickering emulsions of β-carotene were also stabilized with wheat gluten and xanthan gum [153]. The retention of β-carotene was 94 and 70% after one month of storage at 25 and 37 °C, respectively. The emulsions were stable to thermal sterilization. 

Another approach to encapsulate β-carotene was to form solid lipid nanoparticles with palmitic acid crystals as solid shell and corn oil, using whey protein isolate as an emulsion stabilizer [154]. By means of spray-drying and emulsification in flaxseed oil, β-carotene together with eugenol, were encapsulated using octenyl succinic anhydride-modified starches as emulsion stabilizers [155]. In the encapsulated samples, a retention of 71% of β-carotene was reached after 28-day storage at 40 °C.

In another study, nanolipid carriers were used to improve the stability of β-carotene [156]. Hot-high shear homogenization was employed to form the particles, using poloxamer 407 as a surfactant and octyl octanoate and precirol ATO5 as liquid oil and solid lipid, respectively. An encapsulation efficiency of 98% was reached and the particles remained stable after 14-day storage at 25 °C. Another approach to improve nanostructured lipid carriers for β-carotene is the solvent diffusion method [157]. Liquid lipid ratios to total lipids and storage temperature were the most relevant factors affecting β-carotene stability. A particle size of 9.32 nm was achieved, and a β-carotene degradation of 1.9% occurred, with a liquid-to-total lipid ratio of 13.2 and a surfactant concentration of 1.8% after one week of storage at 25 °C.

Soy protein isolate was used as a nanocarrier to increase solubility and enhance the stability of β-carotene [113,158]. The degradation kinetics of β-carotene was assessed during an 80 °C thermal treatment. An improvement in thermal stability was observed in the encapsulated samples [158]. The best results were observed with a ratio of 20 g/kg, in which 70% of β-carotene was retained while being almost completely lost in samples with free β-carotene.

An average encapsulation efficiency of 93% was achieved by the production of bio-based nanoparticles loaded with β-carotene, using the solvent-displacement method with zein and ethylcellulose as encapsulating polymers [159]. A higher bioaccessibility was observed for the zein nanoparticles (37%) compared to ethylcellulose encapsulated particles (8.3%).

The nanoencapsulation of lycopene from tomato peel extracts was also studied [158]. The electrospinning technique was used to fabricate gelatin nanofibers to encapsulate the extracts with an efficiency above 90%. Improved lycopene stability in encapsulated extracts was observed after 14 days of storage at three different temperatures, both in the dark and under exposure to light. 

There are also studies focusing on the encapsulation of lutein to improve its stability and solubility. The solubility of lutein was improved 12-fold by nanoencapsulation using water-soluble chitosan together with poly-γ-glutamic acid [160]. In another study, nanoemulsions were formed using high-intensity ultrasound with whey protein isolate and its polymerized form as stabilizers. Lutein was proven to be stable after 4 weeks of storage at 4 °C when using whey protein isolate with a reduction of only 4%. The emulsion prepared with the polymerized form of whey protein isolate was not stable and separated phases were observed after one week of storage [161]. The encapsulation of lutein in liposomes was also conducted using supercritical CO_2_. High pressure and depressurization rates increased the encapsulation efficiency (up to 97%) and the uniformity of the liposomes. In another study, lipid nanoparticles with encapsulated lutein were improved using fish oil as a carrier [162]. The particles were formed by coupling melting emulsification with high-shear homogenization. The melted lipid phase included lutein, glycerol stearate, carnauba wax, and fish oil. An 88% encapsulation efficiency was achieved, and the lutein in vitro release was improved. 

#### 4.2.2. Effects of Nanoencapsulation on Carotenoid Absorption

The bioavailability of β-carotene dispersed in oil was compared to β-carotene encapsulated in a macroemulsion, nanoemulsion, and nanoparticles, using whey protein isolate as wall material [163]. The absorption of β-carotene in the small intestine of C57BL/6J mice increased from 5.4% (for free β-carotene) to 31, 38, and 59% for macroemulsion, nanoemulsion, and nanoparticles, respectively. β-carotene from the nanoemulsion tended to be converted into retinol and retinyl palmitate and accumulated in the liver while nanoparticles were transported to the circulatory system and stored mostly in adipose tissues. Nanoparticles were formed after the dispersion of whey protein isolate (1%) in water mixed with a dispersion of β-carotene in ethyl acetate (1%) using a speed homogenizer. For the nanoemulsion, a 2% protein solution was made with phosphate buffer and was mixed with 0.1% β-carotene in corn oil (ratio 9:1). 

The bioaccessibility of entrapped β-carotene was also improved by forming an O/W nanoemulsion stabilized with modified starches with different molecular weights. High-pressure homogenization was used to improve the emulsion stability. When compared to β-carotene dispersed in bulk oil, the bioaccessibility was improved from 3.1 to 36%. After 30 days of storage at 4 and 25 °C, the β-carotene retention was higher in the nanoemulsions when compared to the control in all the evaluated conditions [164]. 

The stability and bioaccessibility of encapsulated β-carotene were compared using whey protein isolate and its conjugate with dextran as stabilizers [165]. The use of dextran-conjugated whey protein favored the formation of smaller particles, broader pH stability, higher β-carotene stability, and a controlled release.

Nanoparticles containing β-carotene were formed by using the emulsification-evaporation method with zein-propylene glycol alginate as polymer. A sustained release of β-carotene was observed by means of in vitro simulated gastrointestinal digestion [166]. Similar results were observed when β-carotene was entrapped in whey protein nanoparticles using the pH cycle method and isolated whey protein [167]. Zein nanoparticles with β-carotene were fabricated using carboxymethyl chitosan and tea polyphenols coating using the solvent precipitation method [167]. A controlled release of β-carotene under gastrointestinal simulated conditions was also found. Tea polyphenols were also used for β-carotene encapsulation by means of high-pressure homogenization [168]. An O/W nanoemulsion was prepared with β-carotene in the core oil and polyphenols in the water phase. Tea polyphenols increased the stability of encapsulated β-carotene during storage. Moreover, levels of vitamin A determined in rat liver were positively influenced. The controlled release of β-carotene was also studied in β-lactoglobulin-dextran-conjugated nanoparticles prepared by the homogenization-evaporation method [169]. A good release of β-carotene was observed, and the nanostructures appeared to be more stable against aggregation that may occur at acidic gastric conditions.

The stability of β-carotene encapsulated in lipid nanoparticles using different fats and oils as well as the in vitro digestibility was studied [170]. Olive and corn oil were used as low-melting temperature lipids, and cocoa butter and coconut oil as high-melting temperature lipids. Smaller droplets improved β-carotene stability during storage, while no effect of the lipid type on the carotenoid stability was observed. 

Encapsulation strategies to improve the stability and bioavailability of lycopene were previously summarized [107]. The oral delivery of lycopene was studied in nanoparticles prepared with oligomerized green tea catechin derivative as a carrier, following the nanoprecipitation method [171]. A slower release of lycopene was observed in simulated gastric fluid and a faster release in simulated intestinal fluid. 

An extended release of lycopene was noted in nanocapsules with whey protein [172]. In another study, the bioaccessibility of lycopene of size-different nanoemulsions of tomato extract with 6% lycopene was investigated [173]. The emulsion was prepared using a high-pressure homogenizer. Smaller droplets (<100 nm) showed higher bioaccessibility (0.77%) when compared to droplets between 100 and 200 nm (0.53%) and tomato extracts (0.01%).

Linseed oil was also used to form nanoparticles loaded with lycopene and astaxanthin, applying high-pressure homogenization [174]. Particle size ranged approximately between 200 (100 MPa) to 1200 (5 MPa) nm, depending on the applied pressure. Nanoparticles formed using 100 MPa were only partially digested (66%) and highly bioaccessible (more than 70%). 

The bioaccessibility of astaxanthin was seven-fold higher in nanostructures with potato protein-based carriers [175]. Astaxanthin dissolved in ethanol was added to a potato protein-buffered solution and freeze-dried for particle formation.

Different lutein-containing nanostructures with zein as a stabilizer were compared to evaluate the effects on the physical properties of the structures but also in relation to bioaccessibility [176]. Higher bioaccessibility values were reported for solid lipid particles, followed by nanostructure lipid carriers and nanoemulsions. In another study, the emulsification and solvent evaporation method was used to form nanostructures entrapping lutein, with an efficiency of 81% [177]. Although no change in particle size during 28-day storage at 5, 20, and 40 °C was observed, the lutein content decreased. When compared to conventional emulsions, the lutein cellular uptake by Caco-2 cells was 2.6-fold higher for nanoemulsions. Moreover, postprandial lutein levels after 8 h of a single oral dose were also higher in entrapped lutein when evaluated in mice with an increase of 54% in plasma and liver and 63% in eyes [178]. For this study, lutein was extracted and purified from marigold and encapsulated using low molecular weight chitosan.

## 5. Utilization of Food Processing By-Products

The search for new plant sources of carotenoids to serve as raw materials for food colorants has not been a research priority. Instead, immense attention has been directed to the valorization of agro-industrial by-products, which can provide a huge supply of starting materials for colorants. Numerous reviews and research papers have now been published on this subject. 

Food processing industries, particularly the fruit and vegetable processing industries, generate an enormous amount of by-products, creating a serious operational problem for the industry and posing a threat to the environment [179]. Problems associated with the disposal of these by-products include high handling and transportation costs and limited availability of landfills. Improper disposal increases environmental pollution [180].

Varying from 15 to 50% of the initial weight of the raw materials [181,182], the by-products during fruit and vegetable processing consist mainly of seeds, peels, rinds, pomaces, stems, bagasse, kernels, and husk, containing potentially valuable compounds, such as carotenoids, polyphenols, dietary fiber, vitamins, enzymes, and oils [183]. These phytochemicals can be utilized in different industries, providing economic benefits for industry while helping to mitigate the environmental problems of waste disposal. 

Vegetal by-products are rich sources of natural pigments such as anthocyanins, betalains, carotenoids, and chlorophylls and can meet the demands of natural colorant production at the industrial level for potential food, pharmaceutical, and cosmeceutical applications [179,184,185], Kakar et al. [179] tabulated 60 papers, published during the period 2006–2020, on the utilization of phytochemicals extracted from by-products of various food products.

Utilization of these by-products as raw materials for carotenoid-based natural colorants has been advocated [186,187]. The seeds and peels of carotenogenic fruits and vegetables discarded during processing contain large amounts of carotenoids, usually greater than in the parts retained for processing. Overall, in 12 tropical fruits from Brazil, by-products presented substantially higher carotenoid content than their respective fruit pulps [188].

This valorization of food processing by-products is well exemplified by the tomato processing industry [189,190,191,192,193]. Tomato by-product, which consists of seeds, peels, and small amounts of flesh, are rich in high value-added compounds such as carotenoids and flavonoids. It is used mainly for animal feed or fertilizer or is directly sent to landfill. To reuse fruit and vegetable by-products on an industrial scale, adequate quantities of the by-product and of the extractable high-value ingredients are required. A major industry worldwide, tomato processing is capable of providing ample supply of by-products. Moreover, the tomato peel is about five times richer in lycopene than the pulp [194]. The tomato processing by-product is one of the best sources of lycopene that can be used as a coloring and antioxidant agent [193,195,196,197,198].

Several authors [199,200,201] called attention to the by-products generated by citrus fruit processing. Citrus fruits (e.g., oranges, grapefruits, lemons, limes, tangerines, and mandarins) are among the most cultivated fruits around the globe, widely consumed as processed juices. Citrus-processing industries generate huge amounts of by-products every year; the citrus peel alone accounts for almost 50% of the wet fruit mass [185,202,203]. 

The citrus by-products are repositories of valuable compounds such as flavonoids, dietary fiber, polyphenols, essential oils, ascorbic acid, and sugars, aside from carotenoids [204]. These materials can be transformed into functional food ingredients [179,205]. Mandarin and orange pomaces are equally prominent sources of carotenoids, providing primarily lutein and β-cryptoxanthin. 

Utilization of mango by-products has also been strongly promoted [180,184,206,207]. The by-product of this popular tropical fruit is rich in numerous value-added substances such as dietary fiber, pectin, polyphenols, and carotenoids. 

Méndez-Carmona et al. [192] pointed out possible problems with the exploitation of agri-food by-products: quality of extracted compounds, economic accessibility, industrial reproducibility, and environmental safety. This approach has to take into account the variable quantity and quality of the raw materials, the high costs that might be incurred with their collection and transport, and the extraction, concentration, and purification processes [194].

### 5.1. Green Extraction

A critical step in this valorization effort is extraction. Notably, this research area has rapidly turned to environmentally friendly extraction techniques. Numerous papers have been published on the development and optimization of these methods. A summary of their advantages and disadvantages is shown in Table 1 [196,208,209]. These techniques have gained popularity because they require minimal solvents, are fast and convenient, can increase extraction yield, protect pigments from degradation, enhance the quality of natural colorants, and are eco-friendly [182,210].

Commercial pigment extraction involves some well-established conventional techniques, e.g., extraction with organic solvents assisted by maceration, heating, high hydrostatic pressure or grinding. Although these methods are easy to use, economical, and do not require sophisticated equipment, they consume large volumes of organic solvents, may cause degradation of the pigments, and require long extraction times [210,211]. Organic solvents not only create environmentally hazardous problems, but their residue remaining in the final products also becomes a major safety concern [195].

The environmentally friendly SFE has been a widely employed technique for the extraction of carotenoids. Operating at low temperatures, it avoids thermal degradation. Other advantages are high extraction yields and selectivity, fast extraction rate, absence of solvent and toxic residue in the final product. The most used supercritical solvent, CO_2_ is inert, non-flammable, non-corrosive, nontoxic, and safe for humans and the environment. Disadvantages of this system are high power consumption, very expensive and complex equipment, and elevated operating pressures that could represent a risk for the operators.

Fifteen matrices, including the flesh and peels of sweet potato, tomato, apricot, pumpkin, and peach, as well as flesh and by-products (seeds and stems) of green, yellow, and red peppers, were submitted to SFE by De Andrade Lima et al. [212]. In the vast majority of the matrices tested, the recovery values for α-carotene and β-carotene were higher than 95%.

SC-CO_2_ extraction has been employed to extract lycopene from tomato peel [213], lycopene and β-carotene from industrial tomato peel [214], carotenoids from mango peel [215], lutein from spinach by-products [216], total carotenoid and lycopene from dried tomato pomace [217].

Numerous papers have also been published combining green solvents with innovative extraction techniques, such as UAE, MAE, enzyme-assisted extraction (EAE), PLE, and homogenization. 

A widely explored extracting technique combined with green solvents for the extraction of carotenoids is UAE, an eco-friendly method that allows high efficiency, low energy consumption and equipment costs, high extraction yields, and short extraction time [211,218,219]. Operating at low temperatures, it is suitable for heat-sensitive compounds. 

UAE was employed in the extraction of carotenoids, using as solvents different vegetable oils: sunflower and soy oils with pomegranate peels [220], sunflower oil with peach palm fruit by-products [221], soybean oil with dried peach palm peel [222], canola, corn, and soybean oils with dried pumpkin pulp waste [223], olive oil with orange peel [224], 50% ethanol in water with orange peel [225], olive oil and sunflower oil with passion fruit peel [226], vegetable oils with waste carrot residue [227], soybean and sunflower oils with papaya peel [228], mandarin epicarp [229], and sunflower oil with tomato processing by-product [230].

Pulsed electric field (PEF) processing increases plant cell permeability through electroporation and can be applied in tomato processing to facilitate peeling, increase juice yields, and enhance valorization of tomato by-products [231]. It was used at three different steps of industrial tomato processing. First, it was applied to whole tomatoes to improve peeling, reducing the work required for peel detachment up to 72%. Second, it was applied to chopped tomatoes, increasing tomato juice yield up to 20%. Third, it was used for processing the by-product, comprising of seeds, peels, and a fraction of tomato flesh, to further increase juice yield, the overall yield reaching 90%. The effects on the extraction of high-added value compounds from juicing residues were also studied. Carotenoid extraction yield increased up to 56%. Overall, targeted PEF pretreatments incorporated to industrial tomato processing led to decreased energy demand and increased productivity. 

PEF causes cellular disruption in plant and microbial cells and can therefore be applied to increase intracellular compound extractability. Foods can be processed, maintaining their nutritional and quality characteristics since they are not exposed to high temperatures [232,233]. PEF processing was utilized as pretreatment for tomato peels in order to enhance the extractability of lycopene. PEF treatment at 5 kV/cm improved the carotenoid extraction from tomato peel by 39% as compared with the control extracted with hexane:ethanol:acetone (50:25:25) [232].

Combinations of innovative techniques were also evaluated. Tiwari et al. [234] extracted carotenoids from carrot pomace using ultrasonication and high shear dispersion techniques with flaxseed oil as green solvent. Orange by-product was extracted by combined ultrasonic and enzymatic processes with ethanol as solvent, resulting in higher β-carotene content compared to maceration with ethanol as solvent [235]. 

EA- and HP-assisted extraction of carotenoids, especially lycopene, from tomato by-product using various organic solvents was examined by Strati et al. [236]. Total carotenoid and lycopene extraction yields were increased by the use of pectinase and cellulase enzymes compared to the non-enzyme treated solvent extraction process. Maximum total carotenoid (127 mg/kg DW) and lycopene (89 mg/kg DW) extraction yields were obtained in enzyme-treated samples extracted with ethyl lactate. Strati et al. [236] also showed that the lycopene concentration in the final oleoresin was greatly enhanced by the use of cellulolytic and pectinolytic enzyme preparations. Likewise, Catalkaya and Kahveci [237] reported that oleoresins obtained through pretreatment of industrial tomato waste by a combination of cellulolytic and pectinolytic enzymes followed by ethyl acetate extraction had the highest lycopene recovery.

HP-homogenization was used as a disruption method to recover valuable compounds from tomato peels, using solely water as process medium [194]. The release of water-insoluble lycopene in the aqueous supernatant increased, enabling the recovery of up to 56% of the initial peel content, well above that reported in the literature when using organic solvents or SC-CO_2_.

Two recently described extraction methods are the microemulsion technique and the water-induced hydrocolloidal complexation [192]. The first method allows high extraction yields, stability against oxidation, and reduced use of organic solvents. Some drawbacks are the residual presence of surfactants that could be risky for health and the development of complex systems that may be time-consuming. The second method is characterized by high selectivity and high purity of the extracted compounds, but it needs a subsequent recovery step, and the extraction yields are comparable to that of classical organic solvent extraction.

### 5.2. Biorefinery and Circular Economy Concepts

By-products of fruits and vegetables are considered good candidates for the biorefinery approach [211], which aims at using the same raw material to produce different products. Since these materials are rich not only in carotenoids but also in other bioactive compounds, it has been suggested that they can be transformed into functional food ingredients [179,205].

The recovery of fruit and vegetable processing by-products and their reintegration in the food chain in the form of color, flavor, antioxidant and/or functional food ingredient is in line with the circular economy concept, contributing to a sustainable production system. This is a production and consumption model that involves sharing, reusing, and recycling of products as long as possible, thus minimizing waste [238,239,240]. 

**Figure 3:**
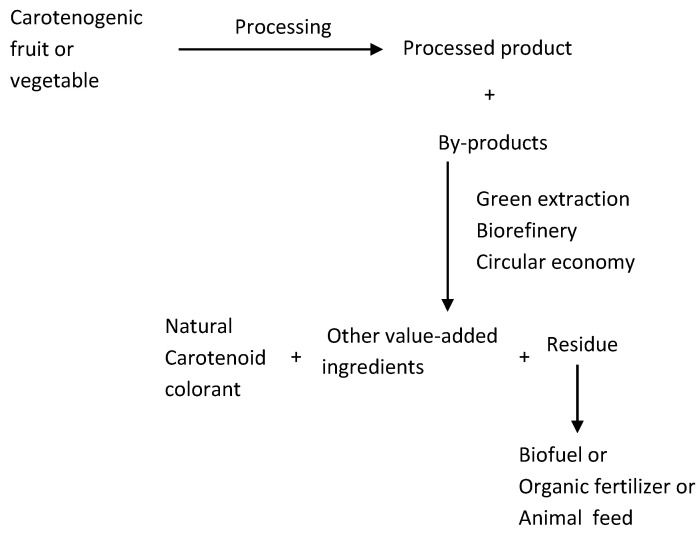
Proposed scheme for the valorization of fruit and vegetable processing by-products, incorporating green extraction, biorefinery, circular economy, and xero waste.

After extraction of value-added ingredients, the residues may be further used for the production of biofuel [241] or organic fertilizer, animal feed, etc. [242], thus contributing to the objective of zero waste (Figure 3).

## 6. Microalgal Carotenoid Colorant

Microbial fermentation for the production of natural colorants has been intensely investigated in recent years [6,7,243,244,245,246,247,248,249]. Several advantages have been cited, such as controlled cultivation, faster growth, higher yields, easier extraction, lower-cost raw materials, no seasonal variations, higher renewability than plant and animal sources, and strain improvement techniques to increase natural pigment. Microbial colorants are often considered better alternative to synthetic food colors compared to higher plant pigments However, in spite of the considerable potential and wide research interest, few carotenoids from microorganisms have reached commercial production: β-carotene by the microalga *Dunaliella salina*, astaxanthin by the microalga *Haematococcus pluvialis*, and β-carotene and lycopene by the fungus *Blakeslea trispora*. According to Begum et al. [250], β-carotene from other microalgae especially *Cyanobacteria* is being produced in large scale in India.

*H. pluvialis* is the widely used scientific name for this microalga. However, Nakata and Ota [251] asserted that the correct name is *H. lacustris*. Ren et al. [252] observed slight phylogenetic distance and genome structural differences between the two *Haematococcus* chloroplast genomes and retained the two names, recommending further studies.

Aside from *D. salina* and *H. fluvialis*, Saini et al. [248] stated that commercial production using carotenoid-rich microalgae, such as *Chlorella zofingiensis* (canthaxanthin), *Scenedesmus* spp. (lutein), *Botryococcusbraunii* (echinenone), and *Phaeodactylum tricornutum* (fucoxanthin), has been established. These colorants, however, are not in the approved lists of FDA, EFSA, and CODEX, presented in Section 7. 

Although in-depth research is still needed to overcome technological bottlenecks, it is widely acknowledged that microalgae can become a prominent and popular source of commercial food pigments in the coming future [253]. Saini et al. [248] highlighted the potential of microalgae, in native or engineered strains, including the metabolic strategies that are used or can be used to produce higher amounts of biopigments. 

Microalgal production of carotenoids has many benefits [7,250,253,254,255,256,257,258]. It is less labor-intensive and only a small area of non-arable land is needed. The growth rate is 5–10 times that of higher plants. It can be carried out year-round and can adapt to a wide range of conditions and climates. Wastewater can be used as a growing medium. Microalgal cultivation may clean the environment through CO_2_ sequestration and wastewater treatment. The large-scale production of carotenoids from microalgae is still limited, however, not yet considered sufficiently cost-effective to compete with chemical synthesis and extraction from plant sources [258,259].

The entire process consists of cell cultivation, biomass harvesting, cell disruption, pigment extraction, purification, and storage. Reviewing the vast literature, the following requirements become evident for the microalgal production of carotenoids to reach the industrial scale:

- Selection of species with appropriate production time and yield of biomass and pigment;
- Efficient culture system design and medium optimization (including the control of operating conditions like temperature, lighting, pH, aeration, agitation, and media components) to maximize biomass and pigment production at low cost.
- Efficient and affordable downstream processes (biomass harvesting, cell wall disruption, pigment extraction, purification, and storage).

Microalgae can be cultivated in open (lakes and ponds) or closed systems (photobioreactors) [260]. The raceway ponds are the cheapest to construct and maintain, consume less energy, and are therefore the most commercially employed. Open ponds, however, have the following disadvantages: nonuniform light intensity, higher evaporation losses, greater requirement for water, reduced temperature control, poor mass transfer rates, diffusion of CO_2_ to the atmosphere, and high risk of contamination [261]. Although much more expensive, photobioreactors are subject to minimal risk of contamination, have better control of culture conditions, require less light and area, and can result in higher biomass and pigment yield [254]. Pigment production in microalgae is affected by various factors such as nutrient availability, salinity, pH, temperature, light wavelength and intensity, photoperiods, pesticides, and heavy metals [250].

Downstream processing, especially cell harvesting and disruption, is difficult. Because of their tiny cell size, microalgal harvesting remains an arduous step and encompasses about 20–30% of the total cost needed for the entire process [262]. It can be performed by filtration, flocculation, centrifugation, sedimentation, and a combination of these strategies [263]. Centrifugation, the most widely employed, is fast, efficient, and suitable for most strains [258]. However, the capital and maintenance costs and energy consumption are too high. Filtration is time and energy-consuming for small-size microalgae. Gravity sedimentation is inexpensive but requires a long time for small, uniformly suspended cells when no additional flocculants are present. Chemical harvesting methods require lower capital investment and consume much less energy but are not as efficient as mechanical methods. Flocculation has received much attention because of the possibility of treating large-scale microalgal suspensions at a lower cost. Technologies are rapidly evolving, including advanced settling tanks, membrane filters, oscillating filters, dissolved air flotation systems, hydrocyclones, electrocoagulation, flow-through centrifuges, and even scalable fractionation [7].

Many microalgal species have rigid cell walls that impede full recovery of the pigments. The efficiency of cell disruption depends on the microalgae species, particularly the cell membrane composition and morphology [264,265]. Mechanical or non-mechanical methods can be used. Mechanical methods consist of bead milling, pressing, high-pressure homogenization, microwave treatment, ultrasonication, autoclaving, and lyophilization [257]. They are best for industry, but energy consumption is high [266]. Non-mechanical methods involve the use of acids or alkalis, osmotic shock, and enzymatic processes. These methods consume less energy and disrupt cell membranes uniformly, but take more time, may affect product quality, and are more difficult to control.

Extraction with organic solvents (e.g., acetone, methanol, ethanol, hexane, dodecane) at a higher temperature and pressure, is popular and has been standardized to meet commercial specifications [258]. This conventional method, however, is known to have inherent limitations: use of large volumes of often toxic solvents, long extraction times, low efficiency and selectivity, and disposal of potentially hazardous solvents to the environment [267,268]. Innovative techniques have been introduced, such as SC-CO_2_ extraction [269,270] and MAE, UAE, EAE, and PLE [196,271]. These techniques have several advantages: extraction of biologically active compounds without degradation or loss of activity [267], use of green solvents (environmentally safe and non-toxic solvents), higher extraction yield, and shorter process time. Poojary et al. [268] reviewed the various methods, pointing out the differences in yield, selectivity, and economic and environmental sustainability.

An option to avoid extraction of carotenoids is to use the whole microalgal biomass if the final application allows (e.g., animal feed) [7]. In addition to carotenoids, microalgal cells can contain other beneficial compounds. The whole biomass would provide additional nutritional benefits even though the carotenoid effect could be diluted by the extra biomass.

### 6.1. β-Carotene

The main carotenoids of microalgae are astaxanthin, β-carotene, lutein, lycopene, zeaxanthin, violaxanthin, and fucoxanthin [255]. The first three are the most studied and are discussed in greater detail in this review.

It is well known that *Dunaliella salina*, the commonly cultivated microalga for β-carotene, has several advantages for commercial production. It lacks a cell wall and produces high levels of β-carotene (10–12% on dry cell weight) [272]. Being halotolerant, it can be cultivated in an open saline mass culture relatively free of competing microorganisms and predators. It is suited for cultivation in coastal areas where seawater is rich in salt and nutrients. Production follows a two-stage strategy. In the first stage, adequate conditions for *D. salina* growth are provided. After cell concentration reaches a certain level, stress conditions (e.g., nutrient limitation, intense light, and low water activity) are applied to accumulate more carotenoids [255].

### 6.2. Astaxanthin

The cost and complexity of synthesizing optically active astaxanthin encouraged the production of microalgal astaxanthin. *H. pluvialis*, the commercial source of microalgal astaxanthin, accumulates up to 3.8% astaxanthin on a dry weight basis [258].

Astaxanthin production by this microalga is much more problematic than the production of *Dunaliella* β-carotene. Since *Haematococus* is a freshwater alga, it is susceptible to contamination with other organisms, making open-air culture extremely difficult. Since the optimal conditions for cell growth differ from those of astaxanthin biosynthesis, a two-stage process is usually adopted [273,274]. The first stage is performed photoautotrophically under controlled culture conditions suitable for microalgal growth, in either tubular, bubble column, or airlift photobioreactors. The following stage, which is less prone to contamination, is performed in open cultivation ponds, subject to environmental and nutrient stress to stimulate carotenoid accumulation.

Although already employed commercially, *H. pluvialis* has slow growth, low biomass yield, high light requirement, and vulnerability to contamination. Numerous studies have been carried out to boost *Haematococcus* astaxanthin production, mainly modifying the design/configuration of the culture system and optimizing the culture conditions, e.g., [275,276,277,278]. Aside from technological improvement, stress must be induced to increase carotenoid concentration, such as nitrogen deprivation, strong light intensity, salt stress, and phosphate deficiency. 

*H, pluvialis* develops a thick and rigid three-layered cell wall during astaxanthin accumulation under stress, which forms a strong barrier to astaxanthin extraction. The methods applied to break the cell wall and extract astaxanthin from *Chlorella* and *Haematococcus* were discussed by Kim et al. [279] comparing efficiency, energy consumption, type and dosage of solvent, biomass concentration, toxicity, scalability, and synergistic combinations. Kim et al. [280] discussed further the various physical, chemical, and biological cell disruption methods and compared the theoretical mechanisms, biomass status (wet, dry, and live), cell-disruption efficacy, astaxanthin extractability, cost, scalability, synergistic combinations, and impact on the stress-sensitive astaxanthin content.

The extraction performance of astaxanthin and lutein from *H. pluvialis* in the red phase (the phase characterized by the transition of the color from green to red) was assessed using bench-scale SC-CO_2_ installation [269]. The cell wall of *H. pluvialis* red biomass was disrupted by mechanical (ball milling) pre-treatment. Maximum recovery of astaxanthin and lutein were 99% and 52%, respectively, at 50 °C and 550 bars.

To determine the processes that yield maximum astaxanthin recovery from *H. pluvialis*, bead milling, high-pressure homogenization, and no disruption of *H. pluvialis* biomass were coupled with spray drying, vacuum drying, and freeze-drying in all possible combinations [281]. Eventually, astaxanthin was extracted using SC-CO_2_. All combinations of milling or high-pressure homogenization and lyophilization or spray-drying resulted in similar recoveries. Evaluating the results in an economic context, bead milling, and spray-drying prior to supercritical CO_2_ extraction were recommended to achieve the maximum astaxanthin recoveries.

In a large-scale investigation of astaxanthin production by *H. pluvialis* in two European cities, it was concluded that for Europe, natural astaxanthin was not a competitive alternative to the synthetic form for aquaculture [282]. However, astaxanthin production by *H. pluvialis* in sites characterized by high solar radiation and high temperatures was considered an attractive venture. Hague et al. [283] reported intensified astaxanthin production using bioethanol wastewater streams as potential ‘green’ media to culture *H. pluvialis*. 

*Chlorella zofingiensis* is considered a good alternative source of astaxanthin for commercial production [284,285]. It has a high growth rate and high cell density that can be achieved through heterotrophic glucose-fed cultivation. 

Reviewing the diversity and distribution of carotenogenic microalgae in Europe, Chekanov [252] cited *Acetabularia acetabulum*, *Botryoccus braunii*, *Bracteacoccus bullatus*, *B. giganteus*, *B. minor*, *Chlainomonas rubra*, *Chloromonas nivalis*, *C. hindakii*, and *C. krienitzii* as potential sources of astaxanthin.

Ambati et al. [258] discussed the in vitro and in vivo trials for the biological activities of astaxanthin: antioxidant effects, anti-lipid peroxidation activity, anti-inflammation, anti-diabetic activity, cardiovascular disease prevention, anticancer activity, immuno-modulation, and neuroprotection. More recent reviews on the health benefits of this highly regarded carotenoid have been published, e.g., [286,287].

### 6.3. Lutein

The potential of microalgae as a lutein source is being intensely investigated. As can be discerned in Section 3, sources of this highly important health-promoting carotenoid are lacking, The commercial source of lutein is the marigold flower. Lutein production from marigold petals, however, has several disadvantages, such as low biomass and low lutein content, high labor demand for harvesting and separation of the petals, season dependence, and arable land occupation. Microalgae have emerged as promising alternatives to marigold. They have a much higher growth rate and can be harvested throughout the year [262,288]. The entire microalgal biomass can be processed for lutein extraction. Microalgae have much higher lutein content compared with marigold and other terrestrial plants. Microalgae processing from harvesting to carotenoid extraction is relatively simple compared with processing marigold flowers, requiring less labor.

Lin et al. [256] compared the different stages of lutein production from marigold flowers and from microalgae. Microalgae had faster growth rates and 3–4 times higher lutein yield. Marigolds needed more land and water but required less nutrients (N, P, K) and less energy.

In spite of the many advantages cited for lutein production by microalgae, a microalgal lutein product has not reached the market. The technical obstacles cited for lutein production by microalgae are lutein values not high enough to be economically feasible on an industrial scale, high harvesting cost, and high energy demand for cell disruption and extraction. Rapid cultivation of algal strains with high lutein content and efficient downstream processing at affordable costs are needed. Lutein productivity can be achieved by selecting adequate species, obtaining high lutein-yielding mutants, and optimizing culture conditions [288,289].

Optimization of culture systems and conditions for enhanced lutein production has been widely pursued, e.g., [288,289,290,291]. Efficient strategies for improving lutein productivity include fed-batch culture, two-stage cultivation, and in situ lutein accumulation [262]. A biomass production process including two stages, heterotrophy/photoinduction, was developed to improve biomass and lutein production by the green microalgae *Tetradesmus incrassatulus* (formerly *Scenedesmus incrassatulus*) [292].

The most investigated factors that affect lutein production are algal species, temperature, light, photoperiod, pH, nutrient availability, and salinity [255,262]. Unlike astaxanthin and β-carotene, which are secondary carotenoids, lutein is a primary carotenoid required for the structure and function of the light-harvesting complexes in photosynthesis. Stress conditions have been repeatedly shown to enhance astaxanthin and β-carotene production but not necessarily lutein accumulation, Augmenting lutein production by stress conditions is difficult [293]. 

Recognizing that the choice of a high-yielding strain, along with an effective photobioreactor design will enable maximal lutein production from microalgae in an economically viable manner, Zheng et al. [294] summarized the recent research progress in lutein production from microalgae, including competent microalgal strains, cultivation systems, and subsequent biomass treatment technologies. 

When cultivated in an outdoor thin-layer photobioreactor, the greatest increase in carotenoid accumulation occurred under conditions of nitrogen sufficiency and high light. The results suggest that in *Tetradesmus* sp., light is a critical factor in the accumulation of carotenoids (mostly lutein) and nitrogen availability plays only a minor role [295]. 

Phototrophic cultivation of microalgae has been considered as a strategy to optimize lutein production due to increased mixing efficiency, higher gas retention time, and shorter cost-effective light path [296]. 

In thermotolerant microalgae *Desmodesmus* sp. F2 and *Coelastrella* sp. F50, cultivated under outdoor tropical conditions, lutein content did not change significantly in microalgae grown with different carbon sources or in different seasons [297]. The major factor influencing productivity was the duration of effective irradiance.

The systematic development of an optimal light-feeding strategy coupled with a semi-continuous mode of reactor operation resulted in greater lutein productivity, photosynthetic efficiency, and CO_2_ fixation rate of *Mychonastes homosphaera* (formerly *Chlorella minutissima*). Moreover, in this process of optimization and integration, light-energy consumption was significantly reduced by 32%, in comparison with constant illumination [289]. 

Light-related (e.g., quality, source, and intensity) strategies have been very effective in promoting the productivity of microalgal biomass and the accumulation of lutein. Chiu et al. [297] observed that in *Tetradesmus* sp., light was a critical factor in the accumulation of carotenoids; nitrogen availability played only a minor role. Optimization of light intensity was also utilized as a means of improving lutein productivity in *Mychonastes* sp. [298] and *Parachlorella* sp. JD-076 [299].

## 7. Regulation and Safety Concerns

### 7.1. Regulation in Different Countries

The use of food colorants, whether derived from natural sources or synthetically produced, is regulated by such agencies as the United States Food and Drug Administration (FDA) and the European Food Safety Authority (EFSA). The EU and a few countries (e.g., China, Korea, India, Japan, and the USA) have legislation to regulate the use of food additives, including color additives [300,301,302]. Many countries have adopted the specifications of Codex Alimentarius or the Joint FAO (Food and Agricultural Organization)/WHO (World Health Organization) Expert Committee on Food Additives (JECFA).

The Food and Drug Administration (FDA) categorizes the color additives approved for use in human foods in two categories [300]:

- Color additives are exempt from batch certification, which includes those derived from fruits, vegetables, plants, or mineral sources. The following natural carotenoids and carotenoid-rich products belong to this category: annatto extract, carrot oil, paprika and paprika oleoresin, saffron, tomato lycopene extract, and tomato lycopene concentrate.
- Color additives are subject to batch certification, which applies to synthetic dyes, lakes, or pigments. There are no carotenoids or carotenoid-rich products in this category.

Colorants are regulated within the EU by Regulation (EC) No 1333/2008 of the European Parliament and of the Council of 16 December 2008 on food additives [301]. They are used to restore the original color of food modified by processing, storage, etc., making food more appealing visually or providing color to colorless foods. Colorants can be natural (extracted from plant, animal, or mineral sources), nature-identical (compounds produced by chemical synthesis), and artificial. The EU relies on the European Food Safety Authority (EFSA) for the safety evaluation of colorants, specifically on the Expert Panel on Food Additives and Flavorings (FAF). Its main roles are [302]:

- To evaluate the safety of new food additives or propose new uses of existing food additives prior to their eventual authorization.
- To re-evaluate all food additives authorized to be used before 20 January 2009.
- To respond to *ad-hoc* requests from the European Commission to review food additives when relevant new scientific information is available and/or to evaluate the change in conditions of use.

The natural carotenoid colorants authorized for food applications in the EU are [303]: paprika extract, capsanthin, capsorubin; annatto bixin; annatto norbixin; tomato lycopene; and *B. trispora* lycopene. 

The following natural carotenoids and carotenoid-containing products can be found in the Codex: annato extracts, annato extracts norbixin-based, β-carotene-rich extract from *D. salina* and from *B. trispora*, lutein from *T. erecta* and as esters from *T. erecta*, lycopene from tomato and from *B. trispora*, and paprika extract [304].

The EU legislation and the Codex Alimentarius specify in detail for each carotenoid the food categories in which they can be used and at what maximum levels [303,304]. These include cheeses, fats and oils, jams, marmalades, processed meat, soups, and spreads, just to mention a few. In the case of the USA, there is less specification. Thus, in relation to uses and restrictions, it is common to indicate “foods generally” and one or two values, if any, about dosage [300].

Mixed carotenes and β-carotene are authorized as food additives in the EU. “Mixed carotenes” refer to two groups of substances, specifically plant carotenes and algal carotenes, whereas “β-carotene” comprises synthetic β-carotene and β-carotene obtained from the fungus *B. trispora*. While the Joint FAO/WHO Expert Committee on Food Additives (JECFA) established in 2001 an ADI (Acceptable Daily Intake) of 0–5 mg/kg bw/day, EFSA concluded that no ADIs for mixed carotenes and β-carotene can be established [305] and that “ exposure to β-carotene from its use as food additive and as food supplement at a level below 15 mg/day do not give rise to concerns about adverse health effects in the general population, including heavy smokers” [306].

A summary of the natural color additives approved for food use by FDA, EFSA, and CODEX is presented in Table 2, making the limited number of permitted natural carotenoid colorants evident. Some regulatory differences can also be easily discerned. Plant-derived lutein is permitted in food in Europe but not in the USA where marigold lutein is allowed only in poultry feed. Saffron is a color additive in the USA but is a spice in Europe. Microbial β-carotene and lycopene are in the EFSA and CODEX lists.

### 7.2. Safety of Carotenoids

Carotenoids have always been part of the diet of humans. The main dietary sources are in general plant-derived foods (mostly fruits and vegetables, but also cereals, herbs, legumes, and oils), although they are also present in animal foods (e.g., seafood, milk and derivatives, egg yolk). Emerging sources are algae, fungi, bactéria, and insects. They are also ingested as food colors and are versatile ingredients for a wide variety of health-promoting and similar products [313,314,315,316,317,318,319].

The intakes of carotenoids vary widely across countries and individuals. As a result of the revision of several studies, it can be accepted that generally the maximum daily intake of individual carotenoids is below 2.5 mg/day, except in the case of β-carotene (1.46–8.80 mg/day), lutein + zeaxanthin (1.00–4.84 mg/day) and lycopene (0.28–10.7 mg/day) (Table 3).

In the 1980s, epidemiological studies, supported by in vitro and animal studies, consistently indicated that β-carotene had a protective effect against cancer. Epidemiological studies in different countries showed an inverse relation between the incidence of cancer, particularly lung cancer, and the dietary intake of β-carotene or the serum level of β-carotene [19]. Since it was recognized that epidemiological studies, which examine the correlation of a food or a food component with the incidence of a disease, do not establish a cause-and-effect relationship, the hypothesis that β-carotene could reduce the risk of cancer was tested in human intervention studies. 

A randomized, double-blind, placebo controlled trial, the α-Tocopherol, β-Carotene Cancer Prevention Study (ATBC Study Group) [320] was carried out in Finland. Male smokers (29,133 participants), 50 to 69 years old, were randomly assigned to four groups, supplemented with: (1) 20 mg β-carotene, (2) 50 mg α-tocopherol, (3) 20 mg β-carotene + 50 mg α-tocopherol, (4) placebo. Supplementation was administered daily for 5–8 years. Instead of reducing the incidence of lung cancer as expected, there was an 18% increase in the groups that received β-carotene. There was no negative or positive effect with α-tocopherol supplementation. 

The unexpected finding was corroborated by a USA study, the CARET study (β-carotene and Retinol Efficiency Trial) [321]. Men and women (18,314 smokers, former smokers or workers exposed to asbestos) were divided into two groups: (1) supplemented daily with 30 mg of β-carotene and 25,000 IU of vitamin A, and (2) placebo. The intervention was supposed to last for 5.5 years, but it was terminated after 3.7 years, 21 months earlier, because the supplemented group had 28% higher incidence of lung cancer than the placebo group. In this and the previous intervention study, supplementation with β-carotene did not affect the incidence of other types of cancer.

Explanations for the unexpected results of the intervention trials had been raised:

- The applied doses of β-carotene in the intervention studies were much higher than physiological doses: 20 mg per day in the ATBC study and 30 mg per day plus 25,000 IU of vitamin A in the CARET study. In the epidemiological studies in which β-carotene intakes were inversely associated with cancer risk, the daily intake of β-carotene was only about 4 mg [322].
- The intervention subjects in the ATBC and CARET studies were mostly smokers and workers exposed to asbestos, thus representing high risk populations. In another large trial, the Physicians’ Health Study, long-term supplementation with β-carotene (50 mg on alternate days for 12 years) produced neither benefit nor harm in terms of lung cancer incidence or overall mortality [323]. Only 11% of the participants were current smokers, thus the study population was at substantially lower risk for lung cancer.
- It was hypothesized that reactive oxygen species of cigarette smoke (or produced as a consequence of asbestosis) in the presence of the relatively high oxygen tension in the lung, induced oxidation of β-carotene, resulting in a prooxidant effect [324].

Eggersdorfer and Wyss [8] argued in their review that the circulating levels of β-carotene in the ATBC and CARET studies were markedly higher as compared to other intervention studies. This indicates that differences in formulation and bioavailability were other factors that could contribute to the divergent results observed.

As an aftermath of these intervention trials and the extensive and intensive discussions about the results around the world, the recommendation to consume carotenoid-containing foods was maintained, but caution was advised, especially to smokers, on the consumption of high-dose supplements [10]. 

Carotenoids are safe compounds, naturally present in the diet of humans all over the world. As an illustrative example, the ADIs for major dietary carotenoids such as lutein or lycopene (Table 3) (1 and 0.5 mg/kg bw/day, respectively, that is 60 mg and 30 mg for a person of 60 kg) exceed by far their daily intakes as ascertained in several studies. Since high supplementation of the provitamin A carotenoid β-carotene has been associated to increased risk of lung cancer in susceptible individuals (heavy smokers, asbestos workers), it has been recommended that its intake remains under 15 mg/day, a quantity that is superior to daily intake reported in diverse studies.

Isolated rare cases of allergenicity linked to the consumption of carotenoid-rich products such as annatto and paprika have been described. It has been concluded, however, that such products are unlikely to represent a safety concern in terms of allergenicity [310,311].

### 7.3. Concern about Contaminants and Safety of Nanomaterials 

The possibility of having residues of toxic solvents, especially when conventional extraction is employed, has been a major concern as discussed above. Plant-derived colorants, being plant extracts, should comply with the pesticide residue legislation. This is an important topic that merits investigation, especially in relation to by-products, considering that pesticide residues were found to be higher in the fruit stalk and near the epidermis (exocarp and fruit receptacle) than in the sarcocarp or pericarp [325]. Moreover, contaminants such as naturally occurring toxins should not be concentrated to amounts of toxicological concern during extraction [326]. 

Bogacz-Radomska and Harasym [23] expressed concern about the accumulation of considerable amounts of heavy metals in algae. Simon et al. [327], on the other hand, cautioned that a significant risk of adulteration of natural colors exists, ranging from simple misbranding or misuse of the term “natural” on a product label to potentially serious cases of physical, chemical, and/or microbial contamination from raw material sources, improper processing methods, or intentional postproduction adulteration.

Amidst the technological success of nanotechnology, as discussed in Section 4, there is growing concern about potential adverse effects (toxicity) of nanomaterials in foods and food packaging. The small size allows nanomaterials easy dispersion and invasion of anatomical barriers in the human body [328]. They can readily pass through the cell membrane to accumulate in the cytosol, affecting cell viability [329,330]. They can travel deeper into the nucleus of cells and damage the DNA, resulting in DNA lesions that can lead to chromosomal aberrations, gene mutations, apoptosis, carcinogenesis or cellular senescence if left unrepaired [331]. Noting that nanoscale materials are naturally present in many commonly consumed foods, such as casein micelles in milk, Mcclements and Xiao [332] opined that many of these nanoparticles are unlikely to have adverse effects on human health but recognized that some of them can have harmful effects. There is general agreement that this topic merits thorough investigation and the use of nanotechnology in foods should be well regulated. 

## 8. Concluding Remarks

Considering their multifaceted roles, carotenoids continue to be intensely studied through the years, changing in terms of the topics investigated. In the last decade, studies on carotenoid colorants derived from higher plants focused on: (1) the replacement of traditional extraction methods with new techniques that are more efficient and environmentally friendly; (2) the use of microencapsulation and nanoencapsulation; and (3) utilization of by-products of the fruit and vegetable processing industry. 

Microalgal production of carotenoids has also been extensively studied with the following objectives: (1) selection of species with appropriate production time and yield of biomass and pigment; (2) efficient culture system and medium optimization, including the control of operating conditions; and (3) efficient and affordable downstream processes.

The impressive scientific and technological advancements, however, have been mostly accomplished at laboratories, needing to be scaled up to the industrial level. There should be tangible and continuous efforts to build bridges between researchers and food processors so that the wealth of knowledge acquired can reach the marketplace.

## Figures and Tables

**Table 1:** Advantages and disadvantages of green extraction techniques.

Technique	Advantages	Disadvantages
Microwave-assisted extraction	Simple, fast, and economical	Can cause thermal degradation and *cis-trans* isomerization of carotenoids
Ultrasound-assisted extraction	Rapid, non-thermal and efficient extraction Low energy consumption and equipment cost	Free radical generation at high sonication power Low selectivity Low ultrasonic power may result in low yield
Pressurized liquid extraction	Fast, requires minimum amount of organic solvent Highly applicable to a laboratory-scale context	Difficult to apply to large volumes due to clogging caused by sugars and pectins of plant matrices
Pulsed electric field extraction	High extraction yield Non-thermal process Low energy use	High cost of instrumentation Bubbles in the samples may cause technical problems
Supercritical fluid extraction	Uses non-flammable, non-toxic and recyclable solvent (CO_2_ and ethanol) Continuous extraction process instead of batch processing Avoids thermal degradation High extraction yield and selectivity	High power consumption High cost of instrumentation
Enzyme-assisted extraction	Rapid and efficient extraction with use of solvents	High cost of the enzymes

References: Saini et al. [196], Majid et al. [208], and Rifna et al. [209].

**Table 2:** Natural color additives approved for food use and Acceptable Daily Intakes when available.

FDA	EFSA (ADI)	CODEX (ADI)
Annatto extract	Annatto bixin (6 mg/kg bw/day) Annatto norbixin 0.3 mg/kg bw/day)	Annatto extract, bixin based (0–12 mg/kg bw) Annatto extract, norbixin based 0–0.6 mg/kg bw)
Paprika and paprika oleoresin	Paprika extract (24 mg/kg bw/day)	Paprika extract (0–1.5 mg/kg bw expressed as total carotenoids)
Tomato lycopene extract and tomato lycopene concentrate	Lycopene from tomato *Blakeslea trispora* lycopene (0.5 mg/kg bw/day)	Lycopene from tomato *Blakeslea trispora* lycopene
Carrot oil Saffron		
	Lutein from different plant sources (1 mg/kg bw/day)	*Tagetes erecta* lutein
	*Dunaliella salina* β-carotene	*Dunaliella salina* (β-Carotene) *Blakeslea trispora* (β-Carotene)

ADI (Acceptable Daily Intake in mg/kg body weight per day). References: USDA [300], European Commission [303], FAO [304], EFSA [307], JECFA WHO [308], EFSA [309,310], JECFA WHO [311], EFSA [312].

**Table 3:** Daily Intake (mg/day) ranges of major carotenoids.

Carotenoid	Range (mg/Day)
α-Carotene	0.14–2.43
β-Carotene	1.46–8.80
β-Cryptoxanthin	0.03–1.36
Lutein + zeaxanthin	1.00–4.84
Lycopene	0.28–10.70
Phytoene	1.89–2.0
Phytofluene	0.47–0.70

Adapted from Meléndez-Martinez [313], Biehler et al. [318], and Olmedilla-Alonso et al. [319].

## Data Availability

Not applicable.
